# How does temperature affect splicing events? Isoform switching of splicing factors regulates splicing of *LATE ELONGATED HYPOCOTYL* (*LHY*)

**DOI:** 10.1111/pce.13193

**Published:** 2018-05-02

**Authors:** Allan B. James, Cristiane P.G. Calixto, Nikoleta A. Tzioutziou, Wenbin Guo, Runxuan Zhang, Craig G. Simpson, Wenying Jiang, Gillian A. Nimmo, John W.S. Brown, Hugh G. Nimmo

**Affiliations:** ^1^ Institute of Molecular, Cell, and Systems Biology, College of Medical, Veterinary, and Life Sciences University of Glasgow Glasgow G12 8QQ Scotland UK; ^2^ Plant Sciences Division, College of Life Sciences University of Dundee Invergowrie Dundee DD2 5DA Scotland UK; ^3^ Informatics and Computational Sciences The James Hutton Institute Invergowrie Dundee DD2 5DA Scotland UK; ^4^ Cell and Molecular Sciences The James Hutton Institute Invergowrie Dundee DD2 5DA Scotland UK

**Keywords:** 5′UTR, alternative splicing, Arabidopsis, circadian, isoform switch, signalling, splicing factors, temperature, thermostat

## Abstract

One of the ways in which plants can respond to temperature is via alternative splicing (AS). Previous work showed that temperature changes affected the splicing of several circadian clock gene transcripts. Here, we investigated the role of RNA‐binding splicing factors (SFs) in temperature‐sensitive AS of the clock gene LATE ELONGATED HYPOCOTYL (LHY). We characterized, in wild type plants, temperature‐associated isoform switching and expression patterns for SF transcripts from a high‐resolution temperature and time series RNA‐seq experiment. In addition, we employed quantitative RT‐PCR of SF mutant plants to explore the role of the SFs in cooling‐associated AS of LHY. We show that the splicing and expression of several SFs responds sufficiently, rapidly, and sensitively to temperature changes to contribute to the splicing of the 5′UTR of LHY. Moreover, the choice of splice site in LHY was altered in some SF mutants. The splicing of the 5′UTR region of LHY has characteristics of a molecular thermostat, where the ratio of transcript isoforms is sensitive to temperature changes as modest as 2 °C and is scalable over a wide dynamic range of temperature. Our work provides novel insight into SF‐mediated coupling of the perception of temperature to post‐transcriptional regulation of the clock.

## Abstract 2

The mechanisms involved in the early responses of plants to temperature remain largely elusive. Here, we investigated the role of RNA‐binding splicing factors (SFs) in temperature‐sensitive alternative splicing of the clock gene LATE ELONGATED HYPOCOTYL. We demonstrate that temperature‐associated isoform switching of SF transcripts is sufficiently rapid and sensitive to temperature changes to contribute to the splicing of the 5′UTR of LATE ELONGATED HYPOCOTYL. This work establishes a role for “splicing of splicing factors” in the coupling of the perception of temperature to post‐transcriptional regulation of the clock.

## 1 INTRODUCTION

Plants respond to daily and seasonal changes in temperature. Although the responsiveness of numerous physiological processes to temperature has been described (McClung & Davis, 2010), the mechanisms involved in the early responses of plants to temperature changes remain elusive. Alternative splicing (AS) is a regulated process producing different mRNA transcripts from precursor messenger RNAs (pre‐mRNAs) of a single gene (Fu & Ares, 2014; Lee & Rio, 2015) and one potential mechanism through which temporal temperature information may be routed is via AS of circadian clock gene transcripts (Calixto, Simpson, Waugh, & Brown, 2016; Filichkin et al., 2010; Filichkin et al., 2015; Filichkin & Mockler, 2012; James, Syed, Bordage, et al., 2012; James, Syed, Brown, & Nimmo, 2012; Kwon, Park, Kim, Baldwin, & Park, 2014; Seo et al., 2012).

Circadian clocks play crucial roles in regulating physiology and behaviour by anticipating environmental changes, principally predictable alterations in light:dark, and concomitant changes in temperature. The framework of the plant circadian clock consists of interlocking gene expression feedback loops (Harmer, 2009; Hsu & Harmer, 2014; Millar, 2016; Nagel & Kay, 2012; Pruneda‐Paz & Kay, 2010), similar in concept to the feedback loops in other eukaryotic clocks (Dunlap, 1999). The central loop of the plant transcriptional core oscillator features a set of two dawn‐expressed, closely related, and partially redundant Myb transcription factors, CIRCADIAN CLOCK ASSOCIATED 1 (CCA1), and LATE ELONGATED HYPOCOTYL (LHY) (Alabadi, Yanovsky, Mas, Harmer, & Kay, 2002; Mizoguchi et al., 2002). Temperature‐dependent AS of *LHY*—in particular cooling associated retention of the 5′UTR intron 1 (I1R, event UAS4 in James, Syed, Bordage, et al., 2012) and the inclusion of exon 5a (event AS5 in James, Syed, Bordage, et al., 2012)—contribute to a notable decline in *LHY* transcript abundance (James, Syed, Bordage, et al., 2012). Mutations in the *SICKLE* (*SIC*) gene, which encodes a nuclear protein implicated in the control of AS, markedly stimulate the accumulation of splice variants of *LHY* and other clock genes at cool temperatures (Marshall, Tartaglio, Duarte, & Harmon, 2016). Moreover, *sic* mutants and mutants in other splicing‐related genes, including for the spliceosomal components *GEMIN2* and *SKIP*, affect the period of the circadian clock (Hernando, Sanchez, Mancini, & Yanovsky, 2015; Jones et al., 2012; Marshall et al., 2016; Sanchez et al., 2010; Schlaen et al., 2015; Wang et al., 2012). Notably, *sic*, *skip*, and *gemin2* mutants are also impaired in temperature compensation, a defining feature of circadian clocks whereby the pace of the clock is largely unaffected across a range of physiologically relevant temperatures (Pittendrigh, 1960).

Although AS of pre‐mRNAs is now strongly associated with the responses of plants to their changing environment (Cui & Xiong, 2015; Filichkin et al., 2015; Reddy, Marquez, Kalyna, & Barta, 2013; Shang, Cao, & Ma, 2017; Staiger & Brown, 2013), less clear is an understanding of the factors influencing co‐transcriptional splice site choice. In this context, the identification of the factors contributing to temperature‐dependent choice of splice sites for *LHY* is therefore of great interest. Polypyrimidine tract‐binding proteins (PTBs) are hnRNPs which bind to the pyrimidine‐rich regions of introns upstream of 3′ splice sites; in general, they have splicing inhibitory functions in both animals and plants (Spellman & Smith, 2006; Wachter, Ruhl, & Stauffer, 2012). PTB‐mediated splicing regulation may involve binding competition between PTB and the splicing factor U2AF65 (Sauliere, Sureau, Expert‐Bezancon, & Marie, 2006; Singh, Valcarcel, & Green, 1995). Pyrimidine‐rich regions are often referred to as polypyrimidine—or pY—tracts and are composed predominantly of uridines (U) (Zamore, Patton, & Green, 1992). In higher eukaryotic systems, the pY varies in length and sequence degeneracy; the mammalian equivalent of U2AF65A recognizes pY tracts with interruptions of cytosines (C) or purines (Jenkins, Agrawal, Gupta, Green, & Kielkopf, 2013; Zamore et al., 1992) and eukaryotic PTB prefers UCUU (or UCUUC) within a larger pY tract (Perez, Lin, McAfee, & Patton, 1997). U2AF65 interacts with U2AF35; they recognize the pY tract and 3′ splice sites, respectively (Moore, 2000), and U2AF65 interacts with SF1 to recruit the U2snRNP in proper assembly of the spliceosome (Berglund, Abovich, & Rosbash, 1998; Zamore et al., 1992). In Arabidopsis, PTB1 and U2AF65 compete for interaction with polypyrimidine‐rich sequences affecting efficiency of splicing (Simpson et al., 2014). Antagonistic interactions between the splicing factor RNA binding motif protein 5 and U2AF65 have also been reported, both in mammalian systems (Bonnal et al., 2008) and in plants (Sugliani, Brambilla, Clerkx, Koornneef, & Soppe, 2010). The Arabidopsis homolog of RNA Binding Motif Protein 5, SUPPRESSOR OF ABI3‐5 (SUA) is involved in the regulated splicing of plant immunity factors (Ding et al., 2014) and some “exitrons” (exon‐like introns), which contain pY (“UCUUCU[U/C]C”) recognition elements for SUA (Marquez, Hopfler, Ayatollahi, Barta, & Kalyna, 2015). Hence, functional interplay between PTB, U2AF65, and SUA may influence the choice of 3′ splice sites.

Three PTB proteins have been identified in Arabidopsis, PTB1‐3 (Stauffer, Westermann, Wagner, & Wachter, 2010; Wang & Brendel, 2004). PTB1 and 2 show a high level of sequence conservation (74% identity at the amino acid level, Stauffer et al. (2010)), whereas PTB3 is more distantly related. PTB1 and 2 are auto‐ and cross‐regulated by AS: one transcript encodes the full‐length protein, the other contains an alternative exon with a premature termination codon, which leads to degradation via nonsense mediated decay (NMD; Stauffer et al., 2010; Wachter et al., 2012). Two U2AF65 genes (*U2AF65A* and *B*) were identified in Nicotiana plumbaginifolia, both bound preferentially to U‐rich sequences and supported splicing in HeLa cell extracts (Domon, Lorkovic, Valcarcel, & Filipowicz, 1998). The Arabidopsis genome possesses four U2AF65 homologues (Wang & Brendel, 2004), although only two of these homologues, U2AF65A and U2AF65B, possess a prototypical U2AF homology motif (Kielkopf, Lucke, & Green, 2004). SUA has an unusual domain structure, possessing two RNA recognition motif domains flanking a zinc‐finger domain (Sugliani et al., 2010).

Here, we demonstrate that PTB and U2AF65A transcripts undergo cold‐induced AS isoform switching (i.e., reversals in abundance of two isoforms) such that the balance between functional and non‐functional transcripts is temperature dependent. Experiments with mutant lines suggest that PTB1‐U2AF65A‐SUA represent part of a network involved in the perception and transduction of prevailing temperature fluctuations to the clock via the splicing of the 5′UTR region of *LHY*. This splicing factor‐*LHY* module is sensitive to temperature changes as modest as 2 °C and is scalable over a wide dynamic range of temperature. We therefore identify some elements of the machinery that links the perception of temperature to the circadian clock and to temperature‐dependent outputs of the clock.

## 2 MATERIALS AND METHODS

### 2.1 Plant material and growth conditions

All plant material was the Columbia (Col‐0) ecotype. The single T‐DNA insertion lines *ptb1‐1* (SALK_013673C) and *ptb2‐*1 (SAIL_736_B12) and the amiRNA knockdown line ami*PTB1&2* (*ami1‐1;2‐1* [Ruhl et al., 2012]) were gifts of Dr. Andreas Wachter (University of Tübingen, Germany). The *sua‐7* (SALK_019773) and *u2af65a‐1* (SALK_075828) mutants (Alonso et al., 2003) were obtained from RIKEN and homozygous mutant lines were selected and characterized. The *smg7‐1 pad4‐1* and *upf1‐3 pad4‐1* double mutants were a gift of Prof. Karel Riha, CEITEC, Brno, Czech Republic. Genotypes of *ptb1‐1*, *ptb2‐1*, ami*PTB1&2*, *sua‐7*, and *u2af65a‐1* were RT‐PCR verified using gene‐ and T‐DNA‐specific primers detailed in Note S3. Seeds were surface sterilized with 3.5% NaOCl and 0.01% Triton X‐100 and washed in sterile distilled H_2_O followed by stratification for 2–3 days in darkness at 4 °C. Plants for RNA‐seq and gene expression analysis were grown hydroponically as described previously (James et al., 2008) in environmentally controlled growth cabinets (Microclima, Snijders Labs, Tilburg, The Netherlands). White light intensity (100 ± 20 μE m^−2^ s^−1^) was provided by Sylvania Grolux F36W/GRO fluorescent tubes, and plants were harvested 5 weeks after sowing (9–13 plants harvested and pooled per condition/time point). Tissue was immediately frozen in liquid nitrogen and stored at −80 °C until further use. For the RNA‐seq diel and temperature time series experiment (Figure S1 and Calixto et al., 2018), plants were harvested at 3‐hr intervals over 24 hr at 20 °C and on Days 1 and 4 after transfer to 4 °C. Transfer to 4 °C was initiated at dusk, unless otherwise stated.

### 2.2 RNA extraction, cDNA synthesis, RT‐PCR, and qPCR

RNA extraction, cDNA synthesis, and qPCR (RT‐qPCR) were performed essentially as described previously (James et al., 2008; James, Syed, Bordage, et al., 2012). Total RNA was extracted with the RNeasy Plant Mini kit (Qiagen) and DNase treated (DNA‐free; Ambion). Complementary DNA (cDNA) was typically synthesized from 2 μg of total RNA using oligo dT primers and SuperScriptII reverse transcriptase (ThermoFisher Scientific). qPCR reactions (1:100 dilutions of cDNA) were performed with Brilliant III SYBR Green QPCR Master Mix (Agilent) on a StepOnePlus (Fisher Scientific‐UK Ltd, Loughborough, UK) real‐time PCR system. The average Ct values for *PP2A* (At1g13320) and *IPP2* (At3g02780) were used as internal control expression levels. The delta‐delta‐Ct algorithm (Livak & Schmittgen, 2001) was used to determine relative changes in gene expression. RT‐PCR was performed using cDNAs and GoTaq Green DNA polymerase (Promega). Products were co‐electrophoresed on 1.5% agarose, 0.5 × TBE gels with 100 bp markers (Roche Diagnostics) and stained with SYBR safe DNA gel stain (ThermoFisher Scientific). All primer sequences are provided in Table S1. Schematics of regions amplified are detailed in Note S2.

### 2.3 High resolution RT‐PCR determination of relative isoform levels

High resolution (HR) RT‐PCR was carried out as described previously (James, Syed, Bordage, et al., 2012; Simpson et al., 2008; Simpson et al., 2016). Total RNA was extracted and cDNA was prepared from three biological replicates. For a gene target, overlapping RT‐PCR primer pairs (each with one 6‐FAM fluorescently labelled primer, see Table S1) were designed to cover the relevant area of gene sequence. RT‐PCR reactions were carried out for 24 cycles as described previously (Simpson et al., 2008), and the products detected on an ABI3730 automatic DNA sequencer along with GeneScan™ 1200 LIZ size standard. Amplicons were accurately sized and mean peak areas calculated using GeneMapper software (Applied Biosystems, Life Technologies). To measure changes in AS of particular isoforms, the peak areas were normalized relative to the peak area values for two reference genes, *UBC21* (At5g25760) and *PP2A* (*PP2AA3*; At1g13320).

### 2.4 RNA‐seq

RNA‐seq libraries were constructed using the Illumina TruSeq library preparation protocol. Libraries had an average insert size of 280 bp and were sequenced three times on the Illumina HiSeq 2500 platform to generate 100 bp paired end reads. After standard quality control and trimming of reads, transcript expression was determined using Salmon version 0.82 (Patro, Duggal, Love, Irizarry, & Kingsford, 2017) in conjunction with AtRTD2‐QUASI (Zhang et al., 2017). Expression profiles for the specific genes used in this study were extracted from the total RNA‐seq dataset of Calixto et al. (2018).

### 2.5 Analysis and presentation of transcript abundance data

Gene and transcript models for *PTB1*, *PTB2*, *U2AF65A*, and *SUA* reported in AtRTD2 (Zhang et al., 2017) are shown in Figure S2. The functional and the main non‐functional premature termination codon‐containing (PTC^+^) *PTB* transcripts are referred to as fully spliced (FS) and alternative exon (AE), respectively. There are two *PTB2* AE isoforms differing in the length of the AE by only three nucleotides (P2 and P4). Two *U2AF65A* transcripts contain a spliced exitron (ID3 and JC2). Two *SUA* transcripts with full coding potential differ by an intron retention event in the 5′UTR. Figure S2 shows details of the individual highly abundant transcripts (low abundance transcripts were removed), whereas other figures have aggregated some transcripts with similar properties (e.g., PTC^+^) for simplicity, as described in the figure legends.

The PTB AE transcripts are targeted for degradation by NMD so their levels detected by qPCR or RNA‐seq underestimate their relative abundance in newly synthesized transcripts prior to NMD. We, therefore, assessed levels of NMD‐degraded transcripts using the strong NMD‐impaired mutant line *upf1‐3* also carrying the *pad4‐1* mutation, which overcomes the lethality of the *upf1‐3* mutation (Riehs‐Kearnan, Gloggnitzer, Dekrout, Jonak, & Riha, 2012; Figure S3a). Measured levels of *PTB* AE transcripts were compensated for NMD by applying the fold increase in abundance in *upf1‐3 pad4‐1* to that in Col‐0 (3.5‐fold for *PTB1* and fivefold for *PTB2*). We present *PTB* transcript data as FS, AE (i.e., not compensated for NMD), and cAE (compensated AE). The *LHY* transcripts from which intron 1 has been excised are referred to as FS; those retaining the intron are referred to I1R. Using the *upf1‐3 pad4‐1* line, we found that I1R transcript levels appear to be relatively insensitive to NMD (Figure S3b) so they are presented without adjustment.

In some figures, *LHY* data is presented as the splice ratio (SpR), defined as FS/(FS + I1R). The SpR at dawn at 20 °C was estimated to be 0.9 from our previous work (James, Syed, Bordage, et al., 2012). *PTB* data are shown as SpR or cSpR (SpR after compensation for NMD), defined as FS/(FS + AE) and FS/(FS + cAE), respectively. The cSpR values for PTB1 and PTB2 at dawn at 20 °C were estimated to be 0.5 and 0.33, respectively, using RNA‐seq data (Figure 2a,b) with AE abundances compensated for NMD. *U2AF65A* has five transcripts: The functional *U2AF65A* transcript is referred to as FS; the other four all introduce PTCs. P2 has retention of intron 11, P3 has an alternative 3′ splice site (Alt3′ss) in exon 11, ID3 has splicing of an exitron, and JC2 has both the Alt 3′ ss and spliced exitron. These PTC^+^ forms have been combined to compare with the protein‐coding isoform and the SpR is defined as FS/(FS + aggregated PTC^+^).

### 2.6 Immunoblotting

Immunoblots for LHY were performed as previously described (James et al., 2008). Plant growth conditions were as for gene expression studies (see Section 2.1). In brief, Col‐0 plants were grown hydroponically and leaf tissue harvested 5 weeks after sowing (9–13 plants harvested and pooled per condition/time point, and tissue immediately flash frozen in liquid N_2_). The anti‐LHY antibody was raised in rabbits against His‐tagged LHY (Kim, Song, Taylor, & Carre, 2003). Two bands specific for LHY protein (James et al., 2008) were quantified using ImageJ software (see legend to Figure S8).

## 3 RESULTS

### 3.1 Temperature‐dependent AS of *LHY*

**Figure 1:**
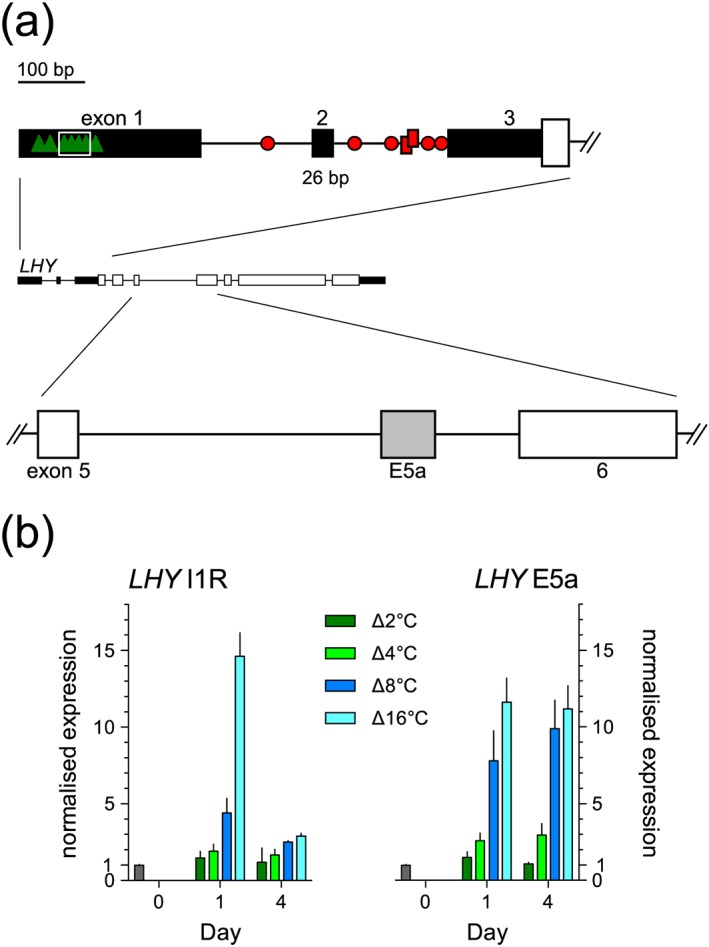
Temperature‐dependent AS of *LHY* transcripts. (a) Gene organization of *LHY* (At1g01060) with the 5′UTR and the region encompassing alternative exon E5a expanded. Solid rectangles, exons; horizontal lines, introns; green triangles, potential SUA consensus binding elements UCUUCUUC, including 4 tandem repeats (white rectangle); red symbols, putative pY regions containing UCUU/UUCU (circles <15 nt, rectangles >15 < 30 nt, see also Note S1). Scale bar is for the expanded models. (b) Levels of I1R transcripts (*left*) and E5a transcripts (*right*) were determined by qPCR using the primers detailed in Table S1. Mature hydroponically grown Arabidopsis Col‐0 plants were grown in 12‐hr light:dark cycles at 20 °C, with cooling to the denoted extent (coloured bars) initiated at dusk. Day 1 plants were harvested 12 hr later at dawn (Day 1) and after a further 72 hr (Day 4). Levels are relative to dawn at 20 °C (grey bars, expression = 1). Bars are means ± SEM, *n* = 2–6

The 5′UTR of *LHY* contains two introns separated by a short mini‐exon of 26 nt (exon 2; Figure 1a). Inspection of the *LHY* 5′UTR region for potential pY‐rich PTB and SUA *cis*‐consensus sequences (“UUCU”; Singh et al., 1995 and “UCUUCU[U/C]C”; Marquez et al., 2015, respectively) showed that intron 2 contains multiple PTB binding sites, whereas intron 1 contains only one, whereas exon 1 contains a region rich in potential SUA binding elements that includes 4 tandem repeats of the sequence “UCUUCUUC” (Figure 1a, Note S1). The plant U2AF65 binding site has not been determined but the RNA recognition motifs 1 and 2 of human U2AF65 bind to variable pyrimidine‐rich sequences to allow recognition of degenerate polypyrimidine tracts (Jenkins et al., 2013). Splicing in the *LHY* 5′UTR region is temperature dependent (James, Syed, Bordage, et al., 2012). Figure 1b shows how inclusion of I1R (event UAS4 in James, Syed, Bordage, et al., 2012) responded to different extents of cooling, either 12 hr or 84 hr after the start of cooling; it increased dramatically on the first day of cooling, reaching up to 50% of the total transcripts (James, Syed, Bordage, et al., 2012), but then recovered. By contrast, inclusion of exon 5a (event AS5 in James, Syed, Bordage, et al., 2012), an alternative exon in the long intron 5 of *LHY*, similarly increased with cooling but persisted with acclimation to the new cooled steady state temperature. The dynamics of this AS event differ from those of I1R (Figure 1b) but the change from E5a skipping to inclusion may involve similar factors. Here, we focus on PTBs, SUA, and U2AF65 as candidate factors in mediating the transient temperature‐associated AS at the *LHY* 5′UTR.

### 3.2 Temperature‐dependent AS of *PTB1*, *PTB2*, *U2AF65A*, *SUA*, and *LHY*

**Figure 2:**
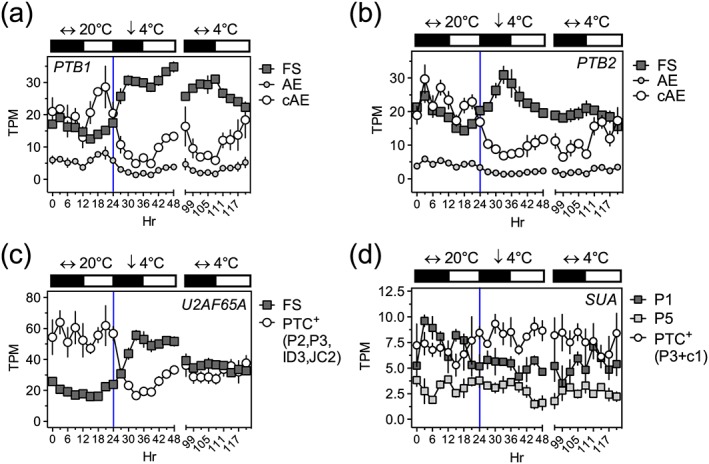
Temperature associated AS and isoform switching of *PTB1*, *PTB2*, *U2AF65A*, and *SUA*. Transcript expression levels from a diel and temperature time series RNA‐seq experiment (see Figure S1) for (a) *PTB1*, (b) *PTB2*, (c) *U2AF65A*, and (d) *SUA*. Black:white bars represent 12‐hr dark:light cycles. Cooling from 20 to 4 °C was initiated at dusk (vertical blue line); TPM, transcripts per million; cAE, AE values compensated for NMD (see Section 2). Data points are means ± SEM, *n* = 3. For panel (c), the PTC^+^ isoform levels are the summed TPMs for the P2, P3, ID3, and JC2 isoforms indicated in Figure S2c. For panel (d), the PTC^+^ isoform levels are the summed TPMs for the P3 and c1 isoforms indicated in Figure S2d. P1 has two spliced 5′UTR introns and is FS. P5 has retention of intron 1 (5′UTR). Isoform switches are seen in a–c [Colour figure can be viewed at http://wileyonlinelibrary.com]

Preliminary analysis by RT‐PCR showed that cooling rapidly affects AS of both *PTB1* and *PTB2* transcripts, giving higher levels of the FS and lower levels of the AE isoforms (Figure S4a). To obtain quantitative data for *PTB1*, *PTB2*, *SUA*, and *U2AF65A*, we examined transcript isoform‐specific expression profiles from an RNA‐seq experiment for Arabidopsis shoot tissue harvested across 3 days before, during, and after a low temperature transition (20 to 4 °C; Figure S1). For all of the genes under study, the majority of introns are efficiently spliced at both 20 °C and 4 °C with AS affecting only specific regions of the genes (Figure S2). The RNA‐seq data for *PTB1* and *PTB2* showed the rapid switching of isoform abundance upon cooling (Figure 2a,b), and qPCR analysis of the same samples for *PTB1* isoforms gave a similar picture to the RNA‐seq profile (Figure S4b). Thus, three approaches—high resolution RT‐PCR, qPCR, and RNA‐seq—all demonstrate the temperature dependence of AS of *PTB* transcripts.

The response of *U2AF65A* to cooling is shown in Figure 2c. The FS and PTC^+^ variants clearly changed in opposite directions within 3 hr of the onset of cooling with a significant increase in the full‐length protein‐coding isoform. For *SUA,* only relatively small changes were observed. Of the *SUA* transcripts with full coding potential, P1 was much more abundant than P5, which retains intron 1 in the 5′UTR (see Figure S2d). P1 declined with cooling, whereas PTC^+^ isoforms P3 and c1 increased with cooling. RNA‐seq analysis of *LHY* transcripts during the cooling time‐course (Figure S5) confirmed the conclusions of our previous work (James, Syed, Bordage, et al., 2012), for example, *LHY* I1R levels increased up to 10–20‐fold with cooling to 4 °C. Thus, AS of *LHY*, *PTB*, *U2AF65A*, and *SUA* transcripts all respond dynamically to cooling.

Because the cooling‐induced abundance of *LHY* I1R transcripts declines after 3 days of exposure to 4 °C (Figures 1b and S5), we assessed the effect of cold adaptation on the AS of the splicing factors. Figure S6 shows that the splice ratios of *PTB1*, *PTB2*, and *U2AF65A* all recover partially after this adaptation period. This is consistent with the view that these factors may be involved in the regulation of transient AS of the *LHY* 5′UTR in response to cooling.

### 3.3 AS of *LHY* and splicing factors is sensitive to both small and brief temperature changes

**Figure 3:**
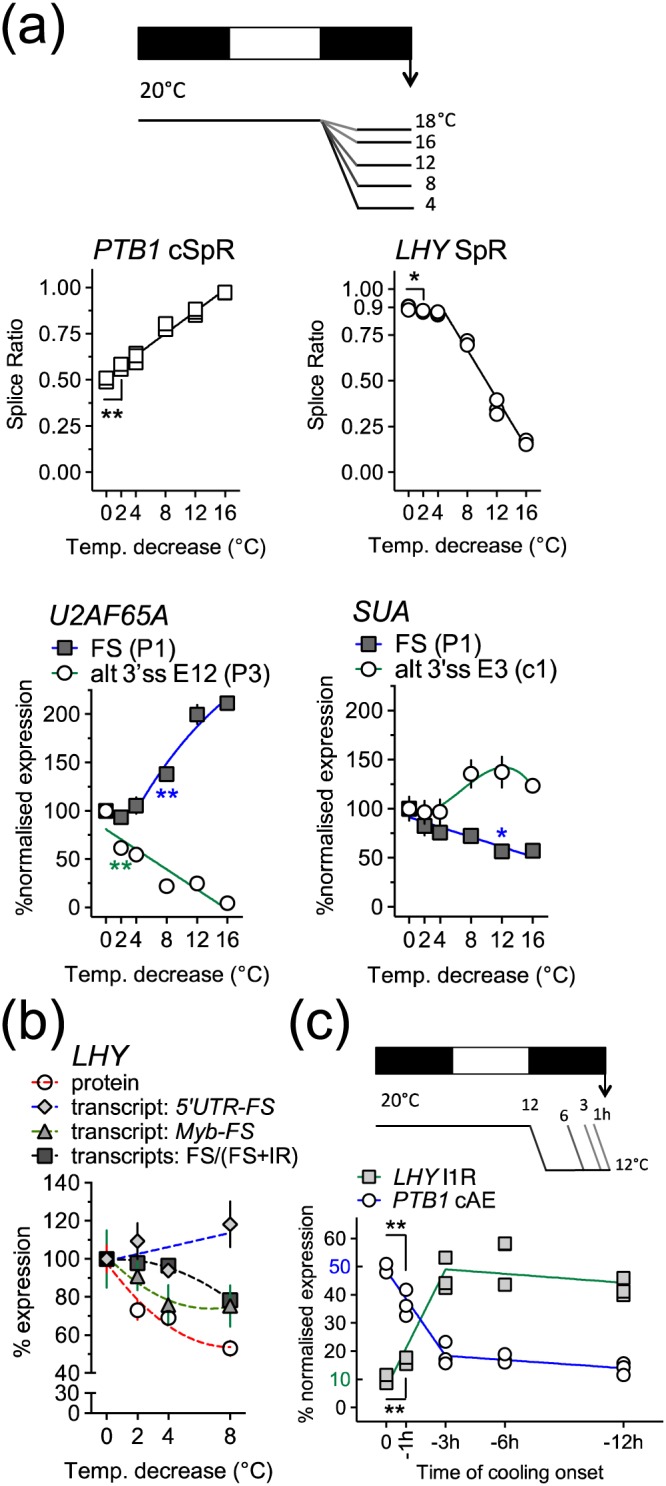
Splicing of *LHY* and splicing factors are sensitive to small extents and durations of cooling. (a) *Upper*; schematic showing experimental regime for examining isoform sensitivity to temperature; plants were grown in 12‐hr light:dark cycles (white and black bars, respectively) at 20 °C with cooling, to the denoted temperature, initiated at dusk and plant tissue harvested at dawn 12 hr later. *Middle*; *PTB1* cSpR and *LHY* SpR and *lower*; *U2AF65A* and *SUA* transcript isoforms. Individual replicates are plotted (*n* = 3) for *middle* plots and means ± SEM (*n* = 3) for *lower* plots. Transcript abundances were measure by qPCR. For *LHY*, the abundances at dawn and 20 °C were taken to represent an SpR of 0.9, and for *PTB1*, they were taken to represent a cSpR of 0.5 (see Section 2); the ratios for other data points were calculated by comparison of the isoform abundances to those at dawn and 20 °C. Only the Δ°C where the change first becomes significant is labelled. For *U2AF65A* and *SUA,* isoform transcripts were expressed relative to the abundances of the forms at dawn and 20 °C. Significant *p* values (unpaired Student's *t* test) are reported for the splice ratios 0 versus Δ2 °C, *p < .05, **p < .01 (b) *LHY* transcripts were measured by qPCR and LHY protein by western blotting (see Figure S8). Values are expressed relative to those observed at dawn and 20 °C, presented as means ± *SD*, *n* = 3, except for LHY protein (mean ± *SD*, *n* = 2). (c) *Upper*; schematic showing experimental regime for examining the responsiveness of splicing to the duration of cooling; plants in 12‐hr light:dark cycles, were cooled from 20 to 12 °C for the denoted duration pre‐dawn. *Lower; LHY* I1R and *PTB1* cAE levels. Transcript abundances were measured by qPCR. The abundance of *LHY* I1R transcripts and PTB1 cAE transcripts at dawn and 20 °C was taken as 10% and 50%, respectively, of the total levels and other values calculated by comparison. Individual replicates are plotted (*n* = 3) and significant *p* values (unpaired Student's *t* test) are reported for 0 versus −1 hr, *n* = 3, **p < .01. Descriptive statistics for best‐fit lines are detailed in Note S4 [Colour figure can be viewed at http://wileyonlinelibrary.com]

We next assessed the sensitivity of these AS events to different extents or durations of cooling. AS of *PTB1* and *LHY* showed remarkable sensitivity to small temperature changes illustrated by modulation of the relative abundance of individual *PTB1* and *LHY* splice isoforms. For example, *PTB1* cAE and *LHY* I1R levels were sensitive to temperature changes of only 2 °C (presented as Δ2 °C; Figure 3a; for clarity, full data with statistical analysis is shown in Figure S7). The *PTB1* cSpR shows a clear linear relationship with temperature; in contrast, the *LHY* SpR responds modestly, though significantly, to temperature transitions of 20–16 °C (Δ4 °C) but much more steeply to changes of 20–12 °C and 20–4 °C (Figures 3a and S7). AS of *U2AF65A* showed similar sensitivity to cooling with the protein‐coding isoform increasing and AS transcript (P3) decreasing significantly at the Δ8 °C and Δ2 °C transitions, respectively (Figure 3a). *SUA* AS was less sensitive to temperature changes (Figure 3a). As shown previously (James, Syed, Bordage, et al., 2012), cooling reduces the expression of LHY protein (Figures 3b and S8) even though the 5′UTR‐FS transcript increases. However, LHY protein does correlate well with the level of *LHY* transcripts covering the Myb domain but not the cold‐induced E5a, which contains a PTC (Figure 3b). This indicates that several AS events contribute to the control of LHY. AS of *PTB1* and *LHY* responded rapidly to cooling with differences in AS detected in plants cooled from 20 to 12 °C (Δ8 °C) for only 1 hr compared with control plants (Figure 3c, for clarity full data with statistical analysis is shown in Figure S9). Thus, specific AS transcript isoforms coded by splicing factor genes are sufficiently rapid and sensitive to cooling that they could comprise part of a primary response network to reductions in temperature.

### 3.4 *LHY* expression and AS is affected in splicing factor mutants

**Figure 4:**
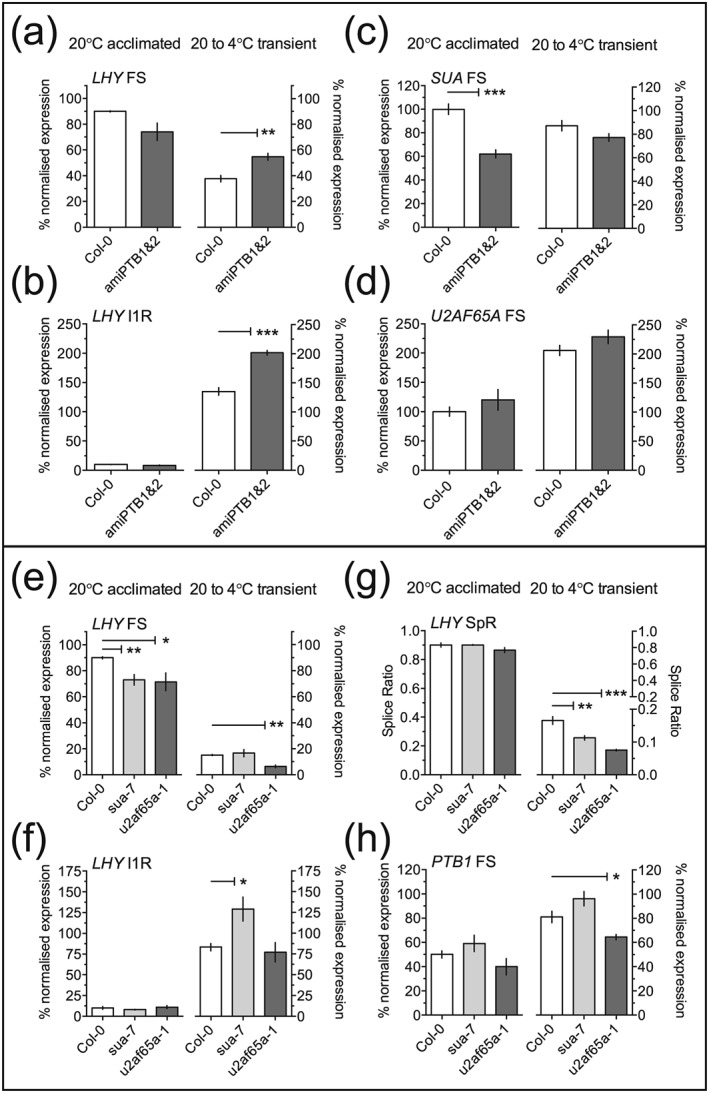
Effects of splicing factor mutations on *LHY* 5′UTR AS. Levels of (a) *LHY* FS, (b) *LHY* I1R, (c) *SUA* FS (P1 isoform), and (d) *U2AF65A* FS (P1 isoform) for Col‐0 and ami*PTB1&2* plants harvested at dawn at ambient temperature (“20 °C acclimated”) or after 12 hr of cooling (“20 to 4 °C transient”), where cooling to 4 °C was initiated at dusk. Levels of (e) *LHY* FS, (f) *LHY* I1R, (g) the *LHY* SpR, and (h) *PTB1* FS for Col‐0, *sua‐7* and *u2af65a‐1* plants harvested at dawn as for (a–d). Expression levels were determined by qPCR. *LHY* FS and I1R values were expressed relative to the values in Col‐0 at dawn and 20 °C taking these to be 90% and 10%, respectively, of total transcripts. *LHY* SpR was calculated as for Figure 3a. *SUA* and *U2AF65A* data were normalized to the values at dawn and 20 °C. *PTB1* FS values were expressed relative to the value in Col‐0 at dawn and 20 °C, taking this to be 50% of total transcripts. Data are means ± SEM, *n* = 3; *p < .05, **p < .01, ***p < .001

To test the hypothesis above, we made use of lines with altered expression of *PTB*s, *U2AF65A*, or *SUA*. First, we found that the single locus mutations *ptb1‐1* and *ptb2‐1* had little effect on *LHY*, *SUA*, or *U2AF65A* splice variants, either at 20 °C or after cooling to 4 °C (data not shown). Although single T‐DNA mutants in *PTB1* and *PTB2* are reported to have no visible phenotype, the double mutant is inviable (Ruhl et al., 2012; Wang & Okamoto, 2009). We, therefore, used an artificial microRNA (ami) knockdown of *PTB1* and *PTB2*, in the Col‐0 background, where the knockdown reduces both mRNAs to around 40% of wild‐type transcript levels (Ruhl et al., 2012). We found that *PTB1* and *PTB2* FS isoform levels were reduced to 47% and 68%, respectively, in the knockdown line compared with Col‐0 (Figure S10). In transiently cooled plants, but not at 20 °C, the levels of both *LHY* FS and I1R transcripts were higher in the amiPTB1&2 knockdown line than in Col‐0 (Figure 4a,b). This suggests that the PTBs can affect levels of *LHY* gene expression, perhaps indirectly, in a temperature‐dependent fashion. *SUA* FS levels were markedly diminished in the amiPTB1&2 line at ambient temperature (Figure 4c, left), but not in transiently cooled plants (Figure 4c, right). We found that *U2AF65A* FS levels were only marginally elevated in the amiPTB1&2 line in both temperature conditions implying that there is either no or only a weak association between U2AF65A and PTB1/2 (Figure 4d).

We extended these analyses by examining AS changes in *sua‐7* and *u2af65a‐1* plants (both in the Col‐0 background; Figure 4e‐h). Both mutations affected the normalized level of *LHY* FS transcripts at 20 °C (Figure 4e, left) without affecting the *LHY* SpR (Figure 4g, left); because little of the I1R transcript is present at 20 °C (Figure 4f, left), the value of this parameter is not very sensitive to small changes in splicing. However, both mutations significantly reduced the *LHY* SpR after transient cooling to 4 °C (Figure 4g, right). The *u2af65a‐1* mutation also had a modest effect on the level of *PTB1* FS transcripts on transient cooling, relative to Col‐0 (Figure 4h). The *sua* mutation had little effect on the expression of *U2AF65A* and vice versa (Figure S11). Overall, the data show that single mutations in *SUA* and *U2AF65A* alter the way that AS of the 5′UTR of *LHY* transcripts responds to temperature.

### 3.5 *PTB1* splicing is regulated by light quantity

**Figure 5:**
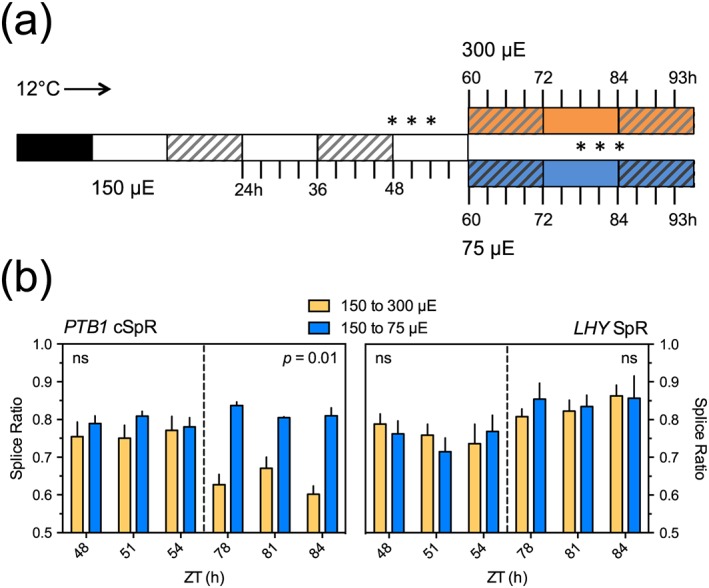
White light intensity consolidates temperature associated AS for *PTB1* but not for *LHY*. (a) Schematic showing experimental regime for examining the effects of light quality on the splice ratios of (b) *PTB1* (left) and *LHY* (right). Plants were subjected to a cool (12 °C) LL regime (light and subjective dark, white and grey hatched bars, respectively). White light quantity was adjusted at 60 hr (from 150 to 300 μE or 75 μE). Samples were harvested within the subjective day before and after the changes in light intensity (denoted by * in the schematic). Extended profiles are provided in Figure S12. Transcript expression levels were measured using qPCR and splice ratios calculated as in Figure 3a. Data are means ± SEM, *n* = 3. *p* value reports significance, separately for the pre‐ and post‐light shifted data, by one‐way analysis of variance, ns; non‐significant (see Note S4) [Colour figure can be viewed at http://wileyonlinelibrary.com]

Because the circadian clock is sensitive to both temperature and light, we next asked whether light quantity could influence the splicing of *PTB1* and *LHY* transcripts. Plants were exposed to constant light at 12 °C for 48 hr in order to allow either increases or decreases in AS to be detected and then shifted to higher or lower light intensities (150 to 300 or 75 μmol.m^−2^.s^−1^; Figure 5a). Increasing the light intensity decreased the cSpR for *PTB1* (Figure 5b), mainly by increasing the level of cAE transcripts (Figure S12a), whereas reducing light intensity modestly increased the cSpR. For *LHY*, the main effect of changing light intensity was on the level of expression of the gene (lower in high light, Figure S12b) and there was no significant effect on *LHY* SpR (Figure 5b). Thus, light intensity affects the splicing of *PTB1*, with lower intensities having similar effects to cool temperatures, although this is not the case for splicing of the 5′UTR of *LHY*.

## 4 DISCUSSION

Recent work has identified temperature‐dependent AS of a number of genes in the Arabidopsis circadian clock and emphasized the importance of these AS events in clock function (reviewed by Romanowski and Yanovsky, 2015). Hence, the nature of the signal transduction mechanism(s) through which temperature changes are detected and how specific splicing events are regulated is of great interest. Cooling associated retention of intron 1 in the 5′UTR of *LHY* is of particular note due to its prevalence with temperature transitions and thus may be synonymous with temperature fluctuations found in nature. AS of *LHY*, therefore, provides a read‐out of the effects of temperature on the plant circadian clock, and we have sought to investigate the relation between this read‐out and the PTB, U2AF65A, and SUA splicing factors.

It is well established that plant PTBs are involved in splicing/AS and are themselves subject to control by AS (Simpson et al., 2014; Stauffer et al., 2010; Wachter et al., 2012). Here, we found that the non‐functional AE forms of the Arabidopsis *PTB*s, which comprise a small proportion of the total transcripts at 20 °C, are reduced even further on cooling. In particular, the relative abundances of the *PTB1* FS and AE isoforms, after compensation for the effects of NMD, show a dramatic isoform switch on cooling to increase the FS protein‐coding isoform after cooling. The functional FS and non‐functional PTC^+^ forms of *U2AF65A* transcripts show a similar pattern. For these three key splicing factors, it is clear that temperature radically affects the levels of functional mRNAs by AS rather than at the level of gene transcription. The splicing of both *PTB1* and *U2AF65A* responded sensitively to cooling, with changes detectable after a transition of only 2 °C, from 20 to 18 °C (Figure 3). The AS of *SUA* was also temperature dependent but responded less sensitively to the extent of cooling. In addition, changes in AS of both *PTB1* and *LHY* were detected after only 1 hr of cooling (Figure 3). The level of sensitivity exhibited by these changes in AS in Arabidopsis approaches that observed in mammals, where a 1 °C change in body temperature is sufficient to induce a concerted splicing switch and changes in splicing are detectable within 30 min of the onset of the temperature change (Preussner et al., 2017).

We provide several lines of evidence that suggest that these rapid and sensitive changes in the splicing of *PTB*s, *U2AF65A*, and *SUA* contribute to the temperature‐dependent splicing of the *LHY* 5′UTR. First, evidence from mutant lines (Figure 4) shows that the effects of the *sua‐7* and *u2af65a‐1* mutations on AS of *LHY* transcripts are dependent on temperature. For example, *LHY* I1R increases in *sua‐7* and *LHY* FS decreases in *u2af65a‐1* at 4 °C thereby contributing to the decrease in *LHY* SpR in the mutants. This shows that both of these factors affect the response of *LHY* 5′UTR splicing to temperature. Knockdown of PTB1 and PTB2 seems to affect the expression level of *LHY* but only after cooling, not at 20 °C. This may account for the decrease in *LHY* levels on cooling (James, Syed, Bordage, et al., 2012 and Figure S5). Second, the sensitivity and speed of the changes in the splicing of *PTB*s and *U2AF65a* seem sufficient to account for changes in the AS of *LHY*. Third, the altered splicing of *PTB1*, *PTB2*, and *U2AF65A* on cooling partially recovered on adaptation to the lower temperature and could therefore account for cold‐induced transient AS changes such as *LHY* I1R. Here, we have focussed on three SFs, but it is quite possible that other factors are involved; For example, splicing of the *PUM23* gene, encoding a PUMILIO RNA‐binding protein, is affected by small temperature changes, which alter the balance between functional and non‐functional transcripts (Streitner et al., 2013). Thus, our data provide another example of combinatorial control of splice site selection in AS, which is already well established in other systems (Barash et al., 2010; Lee & Rio, 2015; Smith & Valcarcel, 2000).

An alternative possibility is transient AS of clock and SF transcripts on cooling results from a failure of some part of the splicing apparatus to respond rapidly to cooling, leading to a transient build‐up of an AS event. We think that this is unlikely, for two reasons. First, we have already ruled out the possibility that some AS events build up because of a transient decline in the capacity for NMD (James, Syed, Bordage, et al., 2012). Second, three different types of transient AS events have been observed (exon skipping in *PRR7*, use of an alternative 3′ splice site in *PRR5*, and intron retention in *TOC1* as well as in *LHY*; James, Syed, Bordage, et al., 2012). It is also possible that temperature‐dependent remodelling of the structure of *LHY* pre‐mRNA contributes to the changes in the abundance of *LHY* I1R on cooling (James, Sullivan, & Nimmo, 2018), but whether this could account for transient effects is less clear.

We have focussed on the *LHY* I1R event as a convenient read‐out in which large changes in the relative abundance of splice variants are observed. The functional effect of this event on LHY protein is not clear but could include effects on translatability (Hinnebusch, Ivanov, & Sonenberg, 2016). The temperature‐dependent inclusion of exon 5a introduces a premature termination codon and leads to NMD, and we have already shown that these two splicing changes together contribute significantly to the reduction in the levels of *LHY* functional transcripts and protein on cooling to 4 °C (James, Syed, Bordage, et al., 2012). The defect in temperature compensation in the *SICKLE* mutant (Marshall et al., 2016) suggests that splicing is involved in this process and hence that the temperature‐dependent splicing of *LHY* may contribute to temperature compensation. In this respect, the changes in *LHY* 5′UTR splicing with temperature are interesting. The *LHY* SpR displays a relationship to temperature akin to a molecular thermostat—it is sensitive to modest changes in temperature, as low as 2 °C, and is also scalable over a wide dynamic range of temperature, both primary considerations of molecular thermometers (McClung & Davis, 2010).

The circadian clock controls many diverse downstream processes and is itself affected by many environmental factors. Here, we also show that changes in light intensity elicit some changes in splicing that are similar to those induced by cooling. AS is also involved in responses of the clock to other biotic and abiotic stresses (Filichkin et al., 2015) and in other signalling pathways such as responses to nutrient deficiency and to ABA (Nishida, Kakei, Shimada, & Fujiwara, 2017; Zhu et al., 2017). Whether these responses involve the same set of splicing factors or different or overlapping splicing networks involved in responses to temperature remains to be investigated and highlights the need to identify plant splicing regulators and their binding sequences.

## AUTHOR CONTRIBUTIONS

A.B.J., C.P.G.C., J.W.S.B., and H.G.N. planned research. A.B.J., C.P.G.C., N.A.T., W.G., W.Y., G.A.N, and C.G.S. performed experiments. A.B.J, C.P.G.C., N.A.T., R.Z., W.G., C.G.S., and J.W.S.B. analysed data. A.B.J., C.P.G.C., J.W.S.B., and H.G.N. wrote the manuscript.

## Supporting information


**Table S1.** Primers used in this study.
**Note S1:** *In silico* pY and SUA binding site analysis of the *LHY* 5′UTR.
**Note S2**: Schematics of primers locations within gene models.
**Note S3**: Genotyping of mutant lines.
**Note S4**: Descriptive statistics for best line fitting.
**Figure S1.** Splicing factor gene structures and RNA‐seq isoform profiles.
**Figure S2.** A temperature and time series RNA‐seq experiment**.**
**Figure S3.** Assessing *PTB1, PTB2* and *LHY* isoform sensitivity to NMD**.**
**Figure S4.** Preliminary characterisation of temperature associated changes in *PTB1* and *PTB2* splicing and confirmation with qPCR**.**
**Figure S5.** The *LHY* FS:I1R isoform switch from the temperature and time series RNA‐seq experiment.
**Figure S6.** Partial recovery of splicing factor isoform switching with cold adaptation.
**Figure S7.** *LHY* and *PTB1* splicing is sensitive to temperature transitions as low as Δ2°C.
**Figure S8.** Cooling reduces the expression of LHY protein.
**Figure S9.** *LHY* and *PTB1* splicing is sensitive to the duration of cooling as low as 1 h.
**Figure S10.** *PTB1* and *PTB2* FS levels in ami*PTB1&2*.
**Figure S11.** Modest changes in reciprocal levels of *SUA* and *U2AF65A* FS levels in *sua‐7* and *u2af65a‐1* mutants.
**Figure S12.** *PTB1* splicing is regulated diurnally and by light quantity.
