# Supplementary material for: How does temperature affect splicing events? Isoform switching of splicing factors regulates splicing of LATE ELONGATED HYPOCOTYL (LHY)
Source: Plant Cell Environ. 2018 May 2;41(7):1539–50. doi: 10.1111/pce.13193 (PMC6033173; doi:10.1111/pce.13193)
Supplement: Supplementary file 1 — Table S1. Primers used in this study. Note S1: In silico pY and SUA binding site analysis of the LHY 5′UTR. Note S2: Schematics of primers locations within gene models. Note S3: Genotyping of mutant lines. Note S4: Descriptive statistics for best line fitting. Figure S1. Splicing factor gene structures and RNA‐seq isoform profiles. Figure S2. A temperature and time series RNA‐seq experiment. Figure S3. Assessing PTB1, PTB2 and LHY isoform sensitivity to NMD. Figure S4. Preliminary characterisation of temperature associated changes in PTB1 and PTB2 splicing and confirmation with qPCR. Figure S5. The LHY FS:I1R isoform switch from the temperature and time series RNA‐seq experiment. Figure S6. Partial recovery of splicing factor isoform switching with cold adaptation. Figure S7. LHY and PTB1 splicing is sensitive to temperature transitions as low as Δ2°C. Figure S8. Cooling reduces the expression of LHY protein. Figure S9. LHY and PTB1 splicing is sensitive to the duration of cooling as low as 1 h. Figure S10. PTB1 and PTB2 FS levels in amiPTB1&2. Figure S11. Modest changes in reciprocal levels of SUA and U2AF65A FS levels in sua‐7 and u2af65a‐1 mutants. Figure S12. PTB1 splicing is regulated diurnally and by light quantity. [file PCE-41-1539-s001.docx]

***Plant, Cell & Environment* Supporting information**

**Article title:** How does temperature affect splicing events? Isoform switching of splicing factors regulates splicing of *LATE ELONGATED HYPOCOTYL* (*LHY*)

**Short running title:** Splicing factor mediated temperature signalling to the clock

**Authors:** Allan B. James, Cristiane P. G. Calixto, Nikoleta A. Tzioutziou, Wenbin Guo, Runxuan Zhang, Craig G. Simpson, Wenying Jiang, Gillian A. Nimmo, John W. S. Brown and Hugh G. Nimmo

The following Supporting information is available for this article:

**Supporting information Notes:**

**Note S1**: *In silico* pY and SUA binding site analysis of the *LHY* 5’UTR.

**Note S2**: Schematics of primers locations within gene models.

**Note S3**: Genotyping of mutant lines.

**Note S4**: Descriptive statistics for best line fitting.

**Supporting information Table:**

**Table S1.** Primers used in this study.

**Supporting information Figures:**

**Figure S1.** Splicing factor gene structures and RNA-seq isoform profiles.

**Figure S2.** A temperature and time series RNA-seq experiment**.**

**Figure S3.** Assessing *PTB1, PTB2* and *LHY* isoform sensitivity to NMD**.**

**Figure S4.** Preliminary characterisation of temperature associated changes in *PTB1* and *PTB2* splicing and confirmation with qPCR**.**

**Figure S5.** The *LHY* FS:I1R isoform switch from the temperature and time series RNA-seq experiment.

**Figure S6.** Partial recovery of splicing factor isoform switching with cold adaptation.

**Figure S7.** *LHY* and *PTB1* splicing is sensitive to temperature transitions as low as Δ2°C.

**Figure S8.** Cooling reduces the expression of LHY protein.

**Figure S9.** *LHY* and *PTB1* splicing is sensitive to the duration of cooling as low as 1h.

**Figure S10.** *PTB1* and *PTB2* FS levels in ami*PTB1&2.*

**Figure S11.** Modest changes in reciprocal levels of *SUA* and *U2AF65A* FS levels in *sua-7* and *u2af65a-1* mutants.

**Figure S12.** *PTB1* splicing is regulated diurnally and by light quantity.

**Note S1**

***In silico* pY and SUA binding site analysis of the 5’UTR of *LHY*.**

Sequences with putative polypyrimidine tract (pY) regions within the intronic regions of Arabidopsis *LHY*. Capitalised sequence: pY regions determined *in silico* using the ASD - Alternative Splicing Workbench (formerly accessed at the EBI web pages; www.ebi.ac.uk/asd-srv/wb.cgi). Mammalian PTBs preferentially bind to UCUU sequences within pY of 15-30 nts ([Ashiya & Grabowski, 1997](#_ENREF_1); [Chan & Black, 1997](#_ENREF_3); [Ruhl *et al.*, 2012](#_ENREF_6)). Potential *Arabidopsis* pY were classified according to length and UCUU/UUCU context. Key to sequence annotations: black, capitalised; short pY regions <15 bp devoid of either UCUU/UUCU sequences. Red, capitalised; pY regions >15 bp with UCUU/UUCU (in either forward or reverse direction) highlighted in red-bold typefont.

*LHY* 5’UTR region pY analysis:

UTR Introns 1 and 2 sequences with 26 bp exon 1 sequence between square brackets [..]. Shaded sequence corresponds to a putative pY that show similarity with the human PTB1 binding site (RBPDB web portal): yellow, blue and green shading correspond to 76, 56 and 50% relative score with hPTB1, respectively.

gtataacagTTTACATTATgagcagtttctaggattcctataacatactaagaTCTCTGTTTggctgctg

agaaacttatagaagcgattaactaaa**TCTT**ATTagctctaaaagttagcataaatgatacgaatctggt

gaTTGATTACTgatatgaagatttgtgaag**[gttttggctgcggtggaTTCGTTTgg]**gtgaggCTTTTGTg

aataataataaagggaa**TTCTT**TTgagttctgctggagaagcagcgactgtttcacggtggactttgaaa

agaT**TTCTCTT**TTgaaTTTCGCTCATCAC**TCTT**A**TCTT**agTGTTTGTggataaaTAT**TTCT**Cataaagta

CT**TTCT**CCTTTgcag

pY with position and sequence (coordinates are relative to the 3’ end of sequence above):

pY (-15, -5) CT**TTCT**CCTTT

pY (-31, -24) TAT**TTCT**C

pY (-45, -39) TGTTTGT

pY (-69, -48) TTTCGCTCATCAC**TCTT**A**TCTT**

pY (-82, -73) T**TTCTCTT**TT

pY (-138, -132) **TTCTT**TT

pY (-163, -157) CTTTTGT

pY (-178, -172) TTCGTTT

pY (-223, -215) TTGATTACT

pY (-268, -262) **TCTT**ATT

pY (-312, -304) TCTCTGTTT

pY (-356, -347) TTTACATTAT

*LHY* 5’UTR region analysis for putative SUA binding sites:

Putative SUA consensus sequence binding sites (‘UCUUCUUC’, ([Marquez *et al.*, 2015](#_ENREF_5))) were found within exon 1 of *LHY*. Exons denoted in red font, introns in mauve. Sequence shown is At1g01060 from the transcriptional start site (-779) to +3.

ttgtggctgagattgcttctggcttctcttcttcttcttccagtcttcttcagcctaaaacagtcttccttcttcttcttcttcttcttcttcttcttcttcttcagttatcttcttccttcttctctctgttttttaaatttatttttagagatttttttttgttttgcttccgatttgattatttccgggaacgatgacttctccggggagttcccggtgagatgataagtcagattgcatacttgtctcctccatggctactctcaagggtataacagtttacattatgagcagtttctaggattcctataacatactaagatctctgtttggctgctgagaaacttatagaagcgattaactaaatcttattagctctaaaagttagcataaatgatacgaatctggtgattgattactgatatgaagatttgtgaaggttttggctgcggtggattcgtttgggtgaggcttttgtgaataataataaagggaattcttttgagttctgctggagaagcagcgactgtttcacggtggactttgaaaagatttctcttttgaatttcgctcatcactcttatcttagtgtttgtggataaatatttctcataaagtactttctcctttgcagtttctctagaatctaaagaggttatcacaacggctttgcaatttgaaaactttcatgtttggggagatcaaagatggtttcttttttatactttacttgttagagaggatttgaagcagcgaatagctgcaccggtcctgttATG

**Note S2**

**Schematics of primer locations within gene models**

Primer sequences are found in Table S1.

| Location of primers: |
| --- |
| *PTB1* RT-PCR:  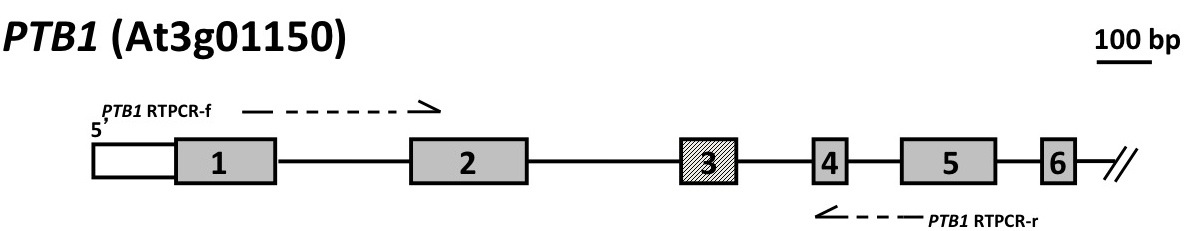  *PTB2* RT-PCR  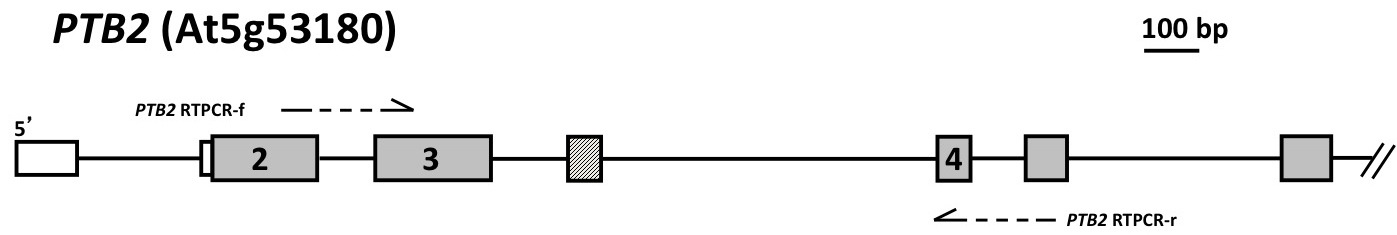  Primers: *PTB1* 296 + 297  *PTB2* 298 +299  *UBC* 156 + 157 |

| *PTB1* qPCR:  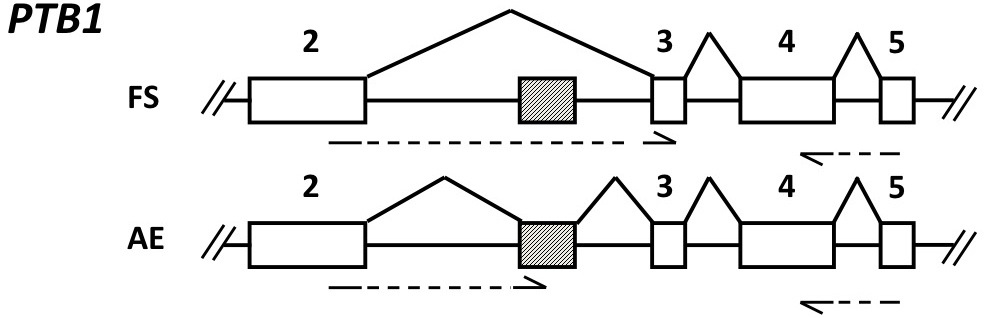 | *LHY* qPCR:  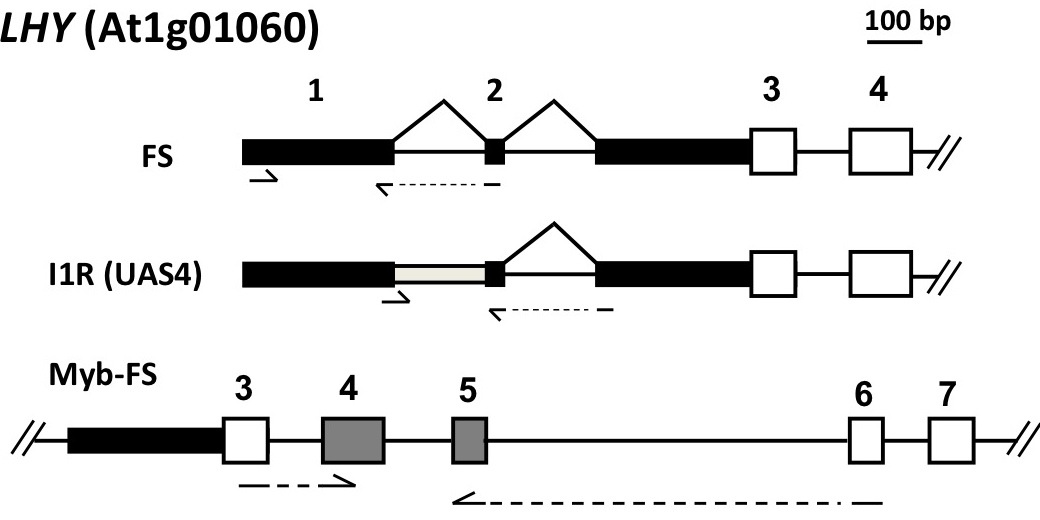 |
| --- | --- |
| Primers:  FS: 540 + 541  AE: 542 + 541 | Primers:  FS (5’UTR): 484 + 485  I1R: 331+ 332  FS (Myb): 141 + 142  E5a: 254 + 255 |

| *SUA* qPCR:  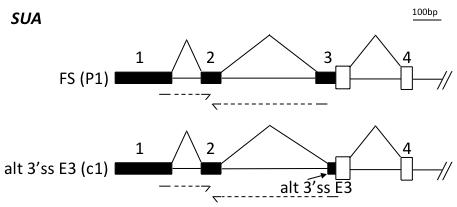 | *U2AF65A* qPCR:  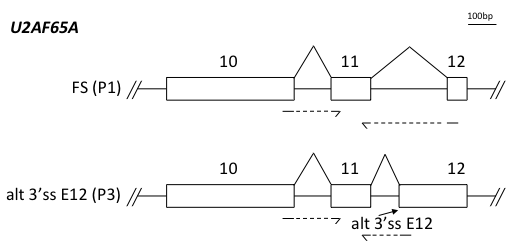 |
| --- | --- |
| Primers:  FS (P1): 533 + 538  alt 3’ss E3 (c1): 533 + 539 | Primers:  FS (P1): 524 + 525  alt 3’ss E12 (P3): 524 + 526 |
|  |  |

**Note S3**

**Genotyping of mutant lines**

**PTB1 and PTB2**

The *PTB1* KO and *PTB2* KO lines (single T-DNA insertion lines *atptb1-1* (SALK_013673C) and *atptb2-1* (SAIL_736_B12), respectively – see cartoon below) and the amiRNA knockdown line ami*PTB1&2* (ami1-1;2-1*)* were a gift of Dr. Andreas Wachter, University of Tuebingen, Germany and have been described elsewhere ([Ruhl *et al.*, 2012](#_ENREF_6)).

|  |
| --- |
| 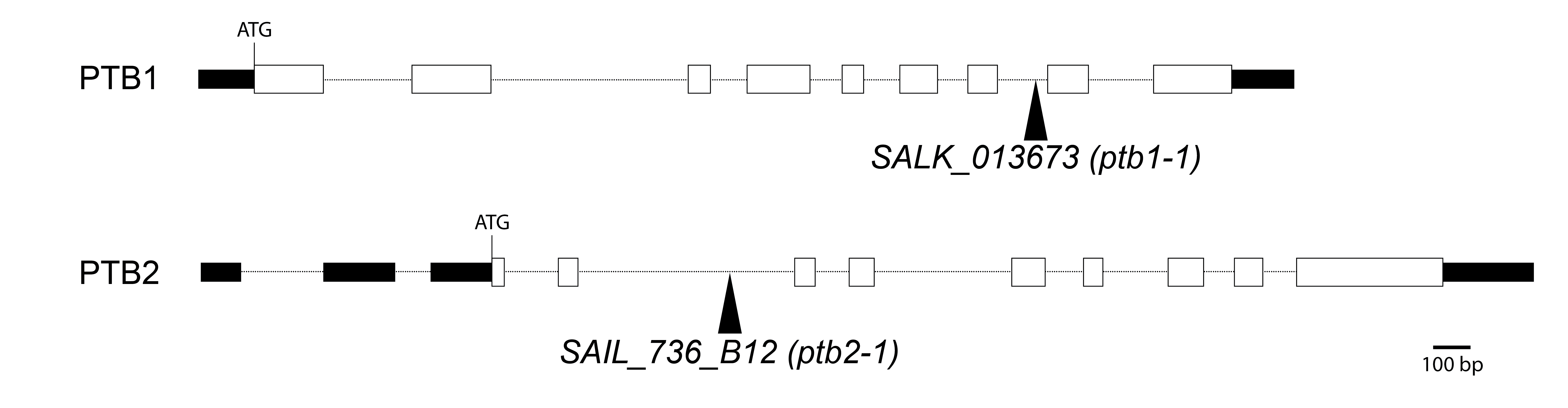 |
| Cartoon depicting assumed locations of T-DNA insertion sites within the *PTB1* and *PTB2* knock-out lines |

|  |
| --- |
| 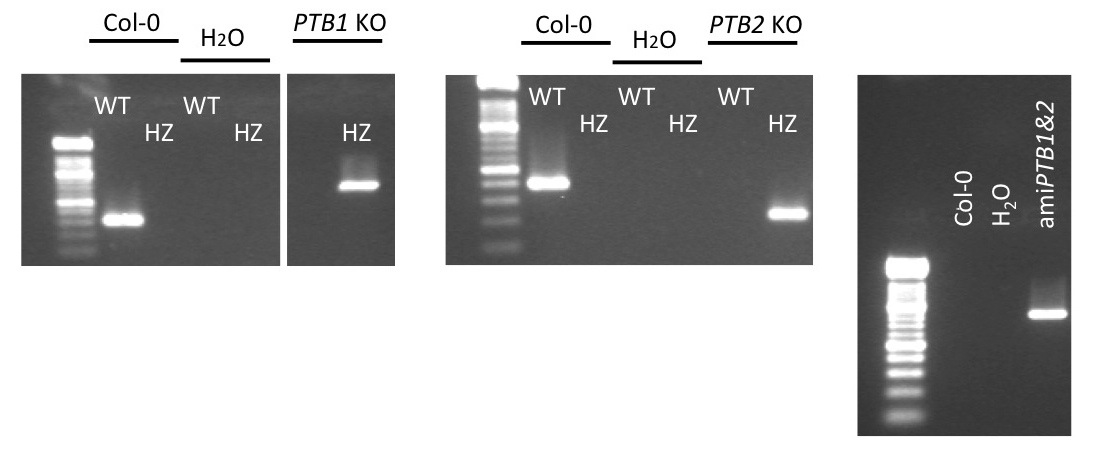 |
| *PTB1* KO, *PTB2* KO and ami*PTB1&2* genotyping was carried out using the following primers, where HZ sets amplify only sequences bearing the T-DNA insertion:  *PTB1* WT: 486 + 487, *PTB1* HZ: 488 +487  *PTB2* WT: 489 + 490, *PTB2* HZ: 491 + 489  ami*PTB1&2*: 497 + 498 |

**SUA**

A *sua* homozygous line (SALK_019773) was identified. The T-DNA insertion occurred within exon 5 (see schematic below). This T-DNA insertion line was not used by ([Marquez *et al.*, 2015](#_ENREF_5)), ([Sugliani *et al.*, 2010](#_ENREF_7)) or ([Zhang *et al.*, 2014](#_ENREF_9)). This new mutant line is named *sua-7* in this study.

|  |
| --- |
| 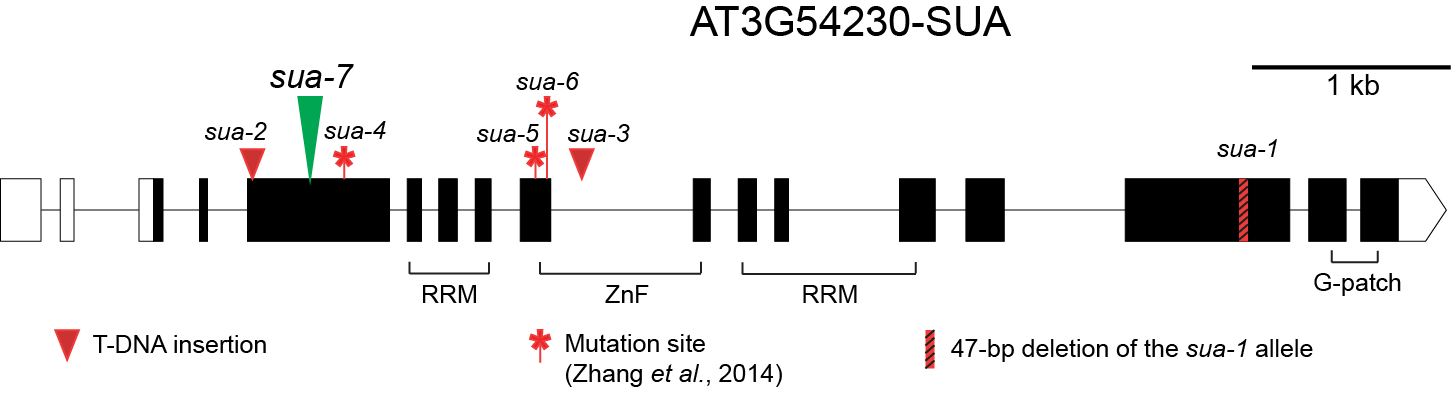 |
| Schematic depicting the assumed location of the T-DNA insertion sites within the *SUA* knock-out line (*sua-7*) in comparison to insertions of other *sua* mutants. |

|  |
| --- |
| 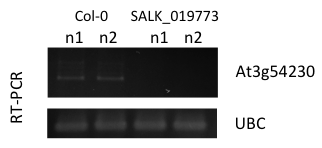 |
| No *SUA* expression was observed in SALK_019773 homozygous line, confirming it is a knockout for *SUA.* Genotyping was carried out using the primers in Table S1. |

**U2AF65A**

A *u2af65a* homozygous line (SALK_019773) was identified. The T-DNA insertion occurred within exon 2 (see schematic below). This line is named *u2af65a-1* in this study.


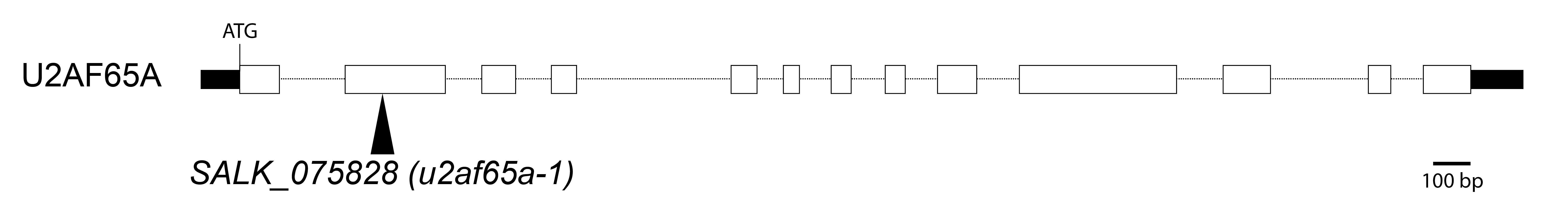


Schematic depicting the assumed location of the T-DNA insertion sites within the *U2AF65A* knock-out line (*u2af65a-1*).


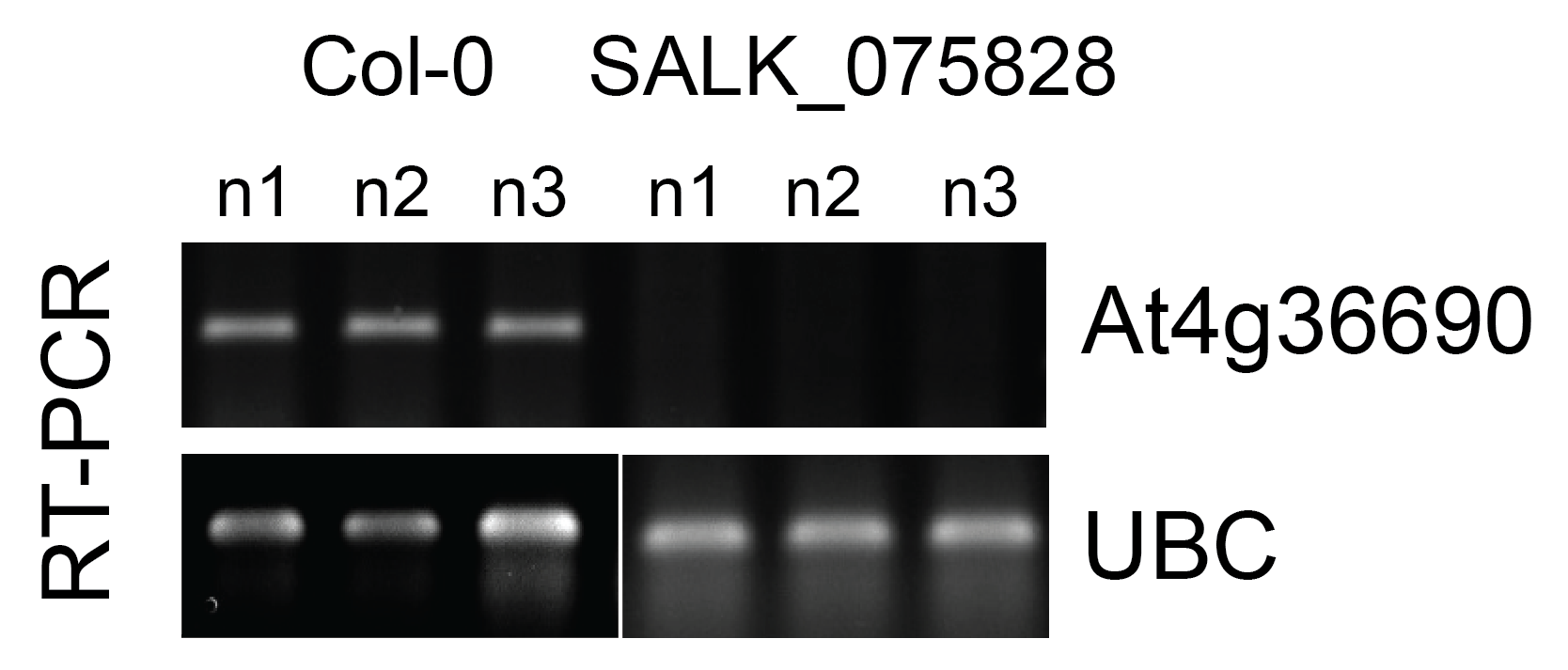


No *U2AF65A* expression was observed in the SALK_075828 homozygous line, confirming it is a knockout for *U2AF65A.* Genotyping was carried out using the primers in Table S1.

**Note S4**

**Descriptive statistics for best line fitting**

Descriptive statistics for Figure 3a: Segmental linear regression was used to fit data points for the *LHY* SpR (*middle right*); one line was used to fit data points with X less than 5.0, and another line points with X greater than 5.0, with the two lines intersecting at X=5.0. Intercept 1 = 0.8958, slope 1= -0.005545, slope 2= -0.06624, *r^2^* = 0.9877.

The relationship between the *PTB1* SpR and temperature decrease (*middle left*) can be predicted from the following linear regression formula; y = 0.02975*x + 0.5131, *r^2^* = 0.9814.

For *U2AF65A* (Figure 3a; *lower left*), plateau followed by one phase association was used for fit the data points for *U2AF65A* FS (blue line), where X0=4.0 was association start and Y0=100 was the average Y value up to time X0=4.0, K (rate constant, expressed as inverse of X scale) = 0.0631, Span (difference between Y0 and Plateau) = 219.7, *r^2^* = 0.9581. The relationship between the *U2AF65A* alt 3’ss E12 (P3) and temperature decrease (*middle left*) can be predicted from the following linear regression formula; y = -5.191*x + 80.91, *r^2^* = 0.8481.

For *SUA* (Figure 3a; *lower right*), the linear regression formula; y = -2.518*x + 91.40, *r^2^* = 0.5831 was used to describe the relationship between *SUA* FS levels and temperature decrease (blue line). A third order polynomial (cubic) line was used to fit points for the alt 3'ss E3 (c1) transcript levels (green line): y = 98.46 -3.584*x + 1.456*x^2 -0.07122*x^3, *r^2^* = 0.4429.

Descriptive statistics for Figure 3c: The relationship between *LHY* I1R levels and duration of cooling (green line) can be described in terms of segmental linear regression constrained with X0=3.0, Intercept 1= 7.090, slope 1= 14.0, slope2= -0.5296, *r^2^* = 0.9042. The *PTB1* cAE (blue line) levels are also modelled as segmental linear regression constrained with X0=3.0, Intercept 1= 48.78, slope 1= -10.13, slope2= -0.4869, *r^2^* = 0.9629.

Descriptive statistics for Figure 5b: *PTB1* SR differed significantly as a function of light intensity, F (5, 12) = 5.079, *p* = 0.0099. Post-hoc Sidak multiple comparison tests comparing, independently, the means of the SRs for the three time points post switch in light quality (78, 81 and 84h) showed that the *PTB1* SR was significantly higher, at the 0.05 level of significance, in low light intensity (75 μE) compared to high light intensity (300 μE) for time points 78h and 84h. The 81h comparison was not significant.

**Table S1.** Primers used in this study

|  |  |  |
| --- | --- | --- |
| **Locus/Primer No.** | **Primer name** | **Primer Sequence (5’-3’)** |
|  |  |  |
| **RT-PCR primers** |  |  |
| ***PTB1*** |  |  |
| 296 | PTB1 RTPCR-f | TTCGTCGAATTTGCTGACTTGAATC |
| 297 | PTB1 RTPCR-r | ATTGAACTAGTGCCTGGAAACCAGC |
| ***PTB2*** |  |  |
| 298 | PTB2 RTPCR-f | GCTTTCATTGAGTTTGAAGATTTGA |
| 299 | PTB2 RTPCR-r | GAACCAGTGCTTGGTATCCGGCTGT |
| ***UBC*** |  |  |
| 156 | UBC-ex2-ex3-f | TTAGAGATGCAGGCATCAAGAGCGC |
| 157 | UBC-ex4-ex3-r | CATATTTCTCCTGTCTTGAAATGAA |
|  |  |  |
| **qPCR primers** |  |  |
| ***LHY* FS (5’UTR)** |  |  |
| 484 | LHY-ex1-f2 | GCTGAGATTGCTTCTGGCTTCT |
| 485 | LHY-ex2-ex1-r | GCAGCCAAAACCCTTGAGAGTA |
| ***LHY* I1R (UAS4)** |  |  |
| 331 | LHY-ex1-int1-f | GGCTACTCTCAAGGGTATAACAGTT |
| 332 | LHY-ex3-ex2-r | GATTCTAGAGAAACCAAACGAATCC |
| ***LHY* FS (Myb)** |  |  |
| 141 | LHY-ex3-ex4-f | GAATTATTAGCTAAGGCAAGAAAGCC |
| 142 | LHY-ex6-ex5-r | GCCTCTTTCTCCAACTTTGTGAAGA |
| ***PTB1* FS** |  |  |
| 540 | PTB1-SPI-f | CGATGTTATCCATCTGGTATTTTCTGC |
| 541 | PTB1-SPI/II-r | TGTGTAATCTCTGCTCCGGTGGGAC |
| ***PTB1* AE** |  |  |
| 541 | PTB1-SPI-f | as above |
| 542 | PTB1-SPII-f | GTTATCCATCTGATGTTGATGCCTC |
| ***U2AF65A* FS (P1)** |  |  |
| 524 | U2ex10-ex11-f | GCTTTTCAGAGGGTTATGTTACAGC |
| 525 | U2ex12-ex11-r | TGGTCAAAGCACCAAACTTTCCGC |
| ***U2AF65A* alt 3’ss E12 (P3)** |  |  |
| 526 | U2altex12-ex11-r | TTATAGCAAAAGGCAAACTTTCCGC |
| 524 | U2ex10-ex11-f | as above |
| ***SUA* FS (P1)** |  |  |
| 533 | SUA-ex1-ex2-f | GTCACCGGTTTCAGCTCATCTTCATC |
| 538 | SUA-ex3-ex2-r | AAGCTCTTTCCTGTGATGAACTG |
| ***SUA* alt 3’ss E3 (c1)** |  |  |
| 533 | SUA-ex1-ex2-f | as above |
| 539 | SUA-altex3-ex2-r | CTTTGATGGTCTGTGATGAACTG |
| ***IPP2* FS** |  |  |
| 499 | IPP2-f | GTATGAGTTGCTTCTCCAGCAAAG |
| 500 | IPP2-r | GAGGATGGCTGCAACAAGTGT |
| ***PP2A*** |  |  |
| 513 | PP2A-f2 | TAACGTGGCCAAAATGATGC |
| 514 | PP2A-r2 | GTTCTCCACAACCGCTTGGT |
|  |  |  |
| **Genotyping PCR** |  |  |
| ***PTB1* locus WT** |  |  |
| 486 | DNA57 | TGGTACATCCGACTGCTGAT |
| 487 | DNA58 | TCTTCCCCTCACAATTGTTTT |
| ***PTB1* locus HZ** |  |  |
| 488 | DNA59 | TGGTTCACGTAGTGGGCCATCG |
| 487 | DNA58 | as above |
| ***PTB2* locus WT** |  |  |
| 489 | DNA60 | GCTATCTTAACTTTTGTCATGCTG |
| 490 | DNA61 | TCCATCGAGGGCAAGTTTTG |
| ***PTB2* locus HZ** |  |  |
| 491 | DNA62 | TTCATAACCAATCTCGATACAC |
| 489 | DNA60 | as above |
| **ami*PTB1&2*** |  |  |
| 497 | AWS1 | CAAGACCCTTCCTCTATA |
| 498 | SL12 | ATTTCACACAGGAAACAG |
| ***SUA* locus WT** |  |  |
| fwd | AT3G54230-F2 | CGATATGGTCGTCAGCAAGA |
| rev | AT3G54230-R2 | TGGTATAGATCTTCTTCTGTTGATTTC |
| ***SUA* locus HZ** |  |  |
| fwd | LBb1.3 | ATTTTGCCGATTTCGGAAC |
| rev | AT3G54230-R2 | as above |
| ***U2AF65A* WT** |  |  |
| fwd | At4g36690-5UTRFw | TCTCCGGTGAGAAAGAGATAGA |
| rev | At4g36690- Ex3Rv | GATGGAGATCGTGACCTTGTT |
| ***U2AF65A* HZ** |  |  |
| fwd | LBb1.3 | as above |
| rev | At4g36690-5UTRFw | as above |
|  |  |  |
| **6-FAM HR RT-PCR** |  |  |
| ***PTB1*** |  |  |
| fwd |  | [6FAM]CCATGAGATTGTTAACAATCAGAGTCC |
| rev |  | CCAGCAGCTTTCTCAAATGTGGC |
| ***PTB2*** |  |  |
| fwd |  | [6-fam] GGATGTTGTGGGGAATGTTCTTCTGG |
| rev |  | GGTATCCGGCTGTCTTCTCGAAAG |
| ***LHY*** |  |  |
| fwd |  | [6-FAM]CCCGGTGAGATGATAAGTC |
| rev |  | CCATCTTTGATCTCCCCAAAC |
|  |  |  |

**Figures S1-S11**

**Figure S1**

**Figure S1.** **A temperature and diel time series RNA-seq experiment.** Three replicate time series experiments were carried out; plants were harvested at the indicated time points (T1-T26). The RNA-seq dataset is also described in [Calixto *et al.* (2018)](#_ENREF_2).

**Figure S2**


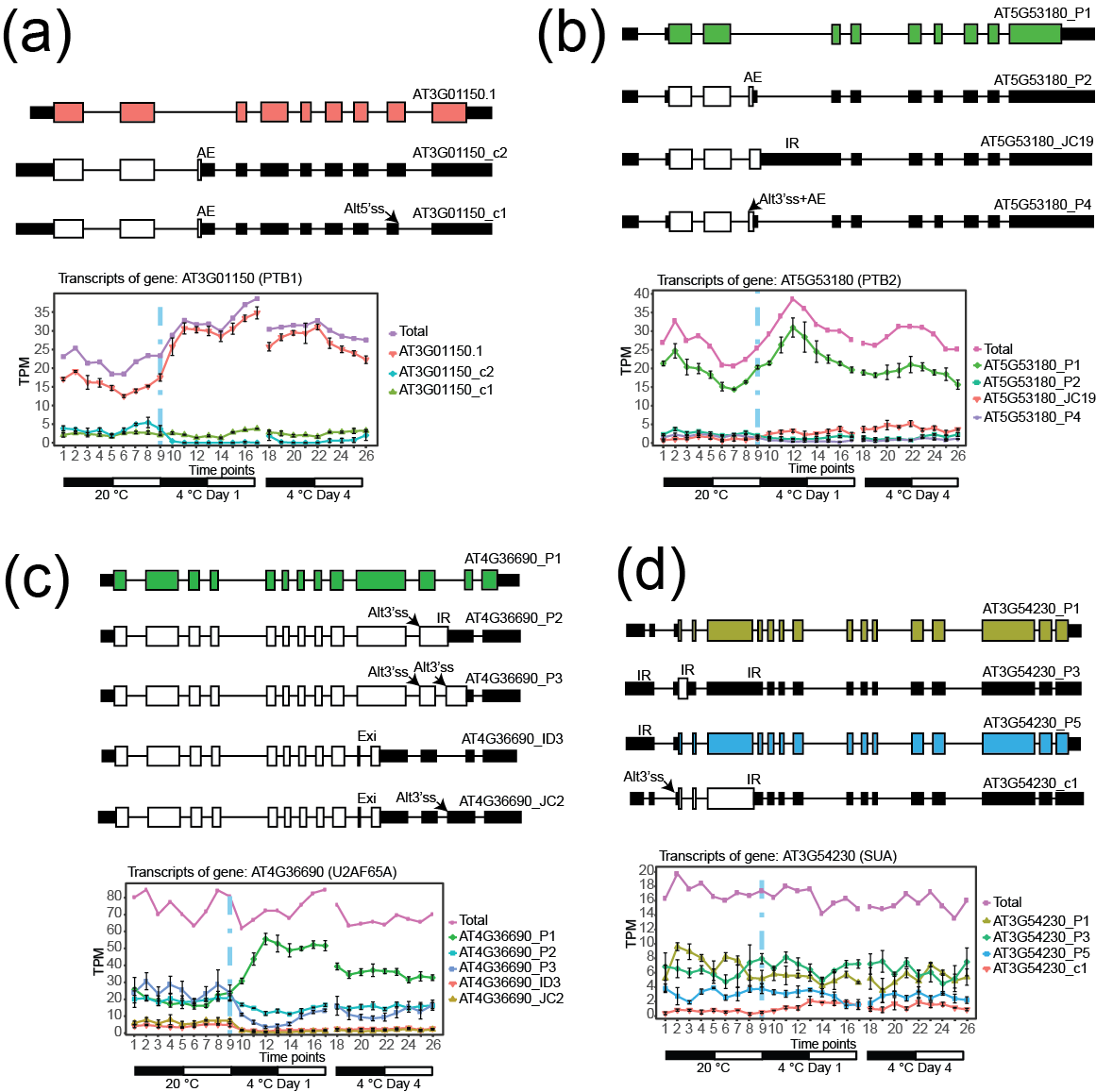


**Figure S2. Splicing factor transcript structures and RNA-seq isoform profiles**

Transcript structures and diel pre- and post- cooling isoform profiles for **(a)** *PTB1*, **(b)** *PTB2*, **(c)** *U2AF65A* and **(d)** *SUA****.*** The y-axes show transcript abundance in transcripts per million (TPM) for a temperature and time series RNA-seq experiment (see Figure S1). Data points are means ± SEM (*n*=3) for the transcript isoforms presented above the plots. Coloured transcripts represent transcripts which code for full-length proteins; white boxes in other transcripts represent the exons forming an open reading frame which terminates prematurely as illustrated by the smaller black boxes (UTR regions). In the expression profiles, cooling was initiated at dusk (dotted vertical blue line at time point 9), black:white bars below expression plots represent 12h dark:light cycles. Gene models were prepared using the Arabidopsis AtRTD2 transcriptome ([Calixto *et al.*, 2018](#_ENREF_2); [Zhang *et al.*, 2017](#_ENREF_8)). AE, alternative exon; IR, Intron Retention; Alt3’ss, alternative 3’ splice site; Exi, exitron.

**Figure S3**

**Figure S3. Assessing *PTB1, PTB2* and *LHY* isoform sensitivity to NMD.** Levels of **(a)** *PTB1* FS and AE (*left*) and *PTB2* FS and AE (58nt) plus AE (61nt) (*right*) and **(b)** *LHY* FS and I1R levels at dawn at 20°C, measured using high resolution (HR) RT-PCR in Col-0 and the NMD-impaired mutants *smg7-1 pad4-1* and *upf1-3 pad4-1.* Data for (a) is mean and ±SEM, *n*=3.

**Figure S4**

**Figure S4.** Preliminary characterisation of temperature associated changes in *PTB1* and *PTB2* AS and confirmation with qPCR**. (a)** Semi-quantitative RT-PCR analysis of *PTB1* and *PTB2* transcripts across a diurnal cycle (12 h LD, white-black bars, respectively) at 20°C and during the first 24 h of cooling to 4°C. FS: fully spliced functional transcripts. AE: alternative exon transcripts (PTC^+^). *UBC* expression (*UBC21;* At5g25760) was used as a loading control. **(b)** Quantitative RT-PCR analysis of *PTB1* transcripts. The FS and cAE abundances for *PTB1* at dawn and 20°C were taken to be equal from Figure 2a; abundances at other time points have been normalised to the dawn, 20°C values of 0.5.

**Figure S5**

**Figure S5. The *LHY* FS:I1R isoform switch from the temperature and time series RNA-seq experiment.** Expression levels from a temperature-diurnal time series RNA-seq experiment (see Figure S1) for *LHY*. Black:white bars represent 12h dark:light cycles. Cooling from 20 to 4°C was initiated at dusk (vertical blue line). I1R values represent the sum of reads corresponding to gene models with intron 1 retained and FS values represent the sum of reads corresponding to models with intron 1 spliced out.

**Figure S6**

**Figure S6. Partial recovery of splicing factor isoform switching with cold adaptation.** Splice ratios for **(a)** *PTB1*, **(b)** *PTB2* and **(c)** *U2AF65A* were calculated from RNA-seq data by averaging the values from the corresponding light time-points 5-9, 13-17 and 22-26 in Figure S1. The values for the AE transcripts of *PTB1* and *PTB2* were compensated for NMD.

**Figure S7**

**Figure S7.** ***LHY* and *PTB1* splicing is sensitive to temperature transitions as low as Δ2°C.** The data summarised in Figure 3(a) for *PTB1* and *LHY* splicing is presented here as bar graphs for **(a)** *PTB1* FS, **(d)** *LHY* FS, **(b)** *PTB1* cAE, and **(e)** *LHY* I1R transcripts, and the resultant ratios for **(c)** *PTB1* and **(f)** *LHY*. Pairwise comparisons were performed *vs* no temperature decrease. Data are means ± SEM, *n*=3 with significant *p* values (unpaired student t-test) reported. *p* value symbols represent the following: ns; not significant, *p* > 0.05; **p* ≤ 0.05; ***p* ≤ 0.01; ****p* ≤ 0.001; **** *p* ≤ 0.0001.

**Figure S8**

**Figure S8.** **Cooling reduces the expression of LHY protein. (a)** Pooled aerial tissue (9-13 plants, representing *n*=1) of mature (5 week old) Arabidopsis Wt (Col-0) maintained in 12 h light:dark cycles (L:D, white:black bars, respectively) at 20°C were harvested at the denoted phase of day (coloured vertical arrows) at either acclimated 20°C, or for a series of cooled plants where cooling to the denoted temperature was initiated at the previous dusk **(b)** Representative western blot of LHY protein levels (bands 2 are specific for LHY, [James *et al.* (2008)](#_ENREF_4)) in response to cooling. Bands 1 and 3 were used as loading controls. Molecular weight markers in kilo daltons (kDa) are denoted.

**Figure S9**

**Figure S9.** ***LHY* and *PTB1* splicing is sensitive to the duration of cooling as low as 1h.** The data summarised in Figure 3(c) for *PTB1* and *LHY* splicing are presented here as bar graphs for **(a)** *PTB1* FS, **(d)** *LHY* FS, **(b)** *PTB1* cAE, and **(e)** *LHY* I1R transcripts, and the resultant ratios for **(c)** *PTB1* and **(f)** *LHY*. Comparisons were performed *vs* the adjacent temperature conditioned sample set. Data are means ± SEM, *n*=3 with significant *p* values (unpaired student t-test) reported. *p* value symbols represent the following: ns; not significant, *p* > 0.05; **p* ≤ 0.05; ***p* ≤ 0.01; ****p* ≤ 0.001; **** *p* ≤ 0.0001.

**Figure S10**

**Figure S10. *PTB1* and *PTB2* FS levels in ami*PTB1&2.*** FS isoform levels for **(a)** *PTB1* and (b) *PTB2* for Col-0 and the double knockdown line (ami*PTB1&2*; Materials and methods) harvested at dawn and 20°C. Values are expressed relative to those for Col-0 plants. Data are means ± SEM *n*=4; ***p*<0.01, ****p*<0.001.

**Figure S11**

**Figure S11. Modest changes in reciprocal levels of *SUA* and *U2AF65A* FS levels in *sua-7* and *u2af65a-1* mutants.** Levels of the **(a)** *U2AF65A* FS isoform in Col-0 and *sua-7* plants and **(b)** *SUA* FS isoform P1 in Col-0 and *u2af65a-1* plants harvested at dawn at ambient temperature (‘20°C acclimated’) or after 12 h of cooling (20 to 4°C transient’), where cooling to 4°C was initiated at dusk. Values are expressed relative to those in Col-0 plants at dawn and 20°C.

**Figure S12**

**Figure S12. *PTB1* splicing is regulated diurnally and by light quantity.** Extended profiles for (**a)** *PTB1* and **(b)** *LHY* splice variants for plants subjected to cold (12°C) LL conditions (see schematic in Figure 5a). White light quantity was adjusted at 60h (150 to 300 μE, or 150 to 75 μE) as indicated by the vertical dotted line. *LHY* expression levels are relative to the values obtained at ZT 27 h in the 150 to 300 μE experiment. The *PTB1* isoform levels are expressed relative to the total (FS + cAE) PTB1 transcripts in a sample taken at dawn in a light:dark cycle at 150 μE and 20°C which was included on the same qPCR plates. *PTB1* cSpRs were calculated as in Figure 3a. Data represents *n*=1, pooled tissue from 10-13 plants except for time points ZT48, 51, 54, 78, 81, and 84 where *n*=3, pooled tissue from 10-13 plants, data points are means ± SEM.

**Supporting information References**

Ashiya M. & Grabowski P.J. (1997) A neuron-specific splicing switch mediated by an array of pre-mRNA repressor sites: evidence of a regulatory role for the polypyrimidine tract binding protein and a brain-specific PTB counterpart. *RNA*, **3**, 996-1015.

Calixto C.P.G., Guo W., James A.B., Tzioutziou N., Entizne J.C., Panter P., . . . ,Brown J.W.S. (2018) Rapid and dynamic alternative splicing impacts the Arabidopsis cold response transcriptome. *bioRxiv*, <https://doi.org/10.1101/251876>.

Chan R.C. & Black D.L. (1997) The polypyrimidine tract binding protein binds upstream of neural cell-specific c-src exon N1 to repress the splicing of the intron downstream. *Mol Cell Biol*, **17**, 4667-4676.

James A.B., Monreal J.A., Nimmo G.A., Kelly C.L., Herzyk P., Jenkins G.I. & Nimmo H.G. (2008) The circadian clock in Arabidopsis roots is a simplified slave version of the clock in shoots. *Science*, **322**, 1832-1835.

Marquez Y., Hopfler M., Ayatollahi Z., Barta A. & Kalyna M. (2015) Unmasking alternative splicing inside protein-coding exons defines exitrons and their role in proteome plasticity. *Genome Res*, **25**, 995-1007.

Ruhl C., Stauffer E., Kahles A., Wagner G., Drechsel G., Ratsch G. & Wachter A. (2012) Polypyrimidine tract binding protein homologs from Arabidopsis are key regulators of alternative splicing with implications in fundamental developmental processes. *Plant Cell*, **24**, 4360-4375.

Sugliani M., Brambilla V., Clerkx E.J., Koornneef M. & Soppe W.J. (2010) The conserved splicing factor SUA controls alternative splicing of the developmental regulator ABI3 in Arabidopsis. *Plant Cell*, **22**, 1936-1946.

Zhang R., Calixto C.P.G., Marquez Y., Venhuizen P., Tzioutziou N.A., Guo W., . . . ,Brown J.W.S. (2017) A high quality Arabidopsis transcriptome for accurate transcript-level analysis of alternative splicing. *Nucleic Acids Res*, **45**, 5061-5073.

Zhang Z., Liu Y., Ding P., Li Y., Kong Q. & Zhang Y. (2014) Splicing of receptor-like kinase-encoding SNC4 and CERK1 is regulated by two conserved splicing factors that are required for plant immunity. *Mol Plant*, **7**, 1766-1775.
